# Mathematical modeling of the glucagon challenge test

**DOI:** 10.1007/s10928-019-09655-2

**Published:** 2019-09-30

**Authors:** Saeed Masroor, Marloes G. J. van Dongen, Ricardo Alvarez-Jimenez, Koos Burggraaf, Lambertus A. Peletier, Mark A. Peletier

**Affiliations:** 1 Maastricht University, Maastricht, The Netherlands; 2 Netherlands Cancer Institute, Amsterdam, The Netherlands; 3 Amsterdam University Medical Center, Amsterdam, The Netherlands; 4 Centre for Human Drug Research, Leiden, The Netherlands; 5 Leiden University, Leiden, The Netherlands; 6 Eindhoven University of Technology, Eindhoven, The Netherlands

**Keywords:** Glucagon, Insulin, Glucose, Receptor internalization

## Abstract

A model for the homeostasis of glucose through the regulating hormones glucagon and insulin is described. It contains a subsystem that models the internalization of the glucagon receptor. Internalization is a mechanism in cell signaling, through which G-protein coupled receptors are taken from the surface of the cell to the endosome. The model is used to interpret data from a glucagon challenge test in which subjects have been under treatment with a novel glucagon receptor anti-sense drug which is aimed at reducing the number of receptors in the liver. It is shown how the receptor internalization results in tolerance of the blood glucose concentration to glucagon-induced hyperglycemia. We quantify the reduction of the number of receptors using the model and the data before and after treatment.

**Electronic supplementary material:**

The online version of this article (doi:10.1007/s10928-019-09655-2) contains supplementary material, which is available to authorized users.

## Introduction

Insulin and glucagon are two major hormones in the regulatory system of blood glucose. Insulin is secreted by the pancreas, especially when the concentration of blood glucose is high. It promotes the uptake of glucose from the blood into tissues. Glucagon is a hormone that has an impact on glucose–insulin homeostasis through its receptor. It is secreted by the pancreas when levels of blood glucose are low and facilitates the release of glucose from its glycogen storage in the liver into the bloodstream.

Four decades ago Bergman et al. [1] developed one of the first mathematical models for glucose regulation. With this model it became possible to estimate the insulin sensitivity of diabetic patients. Also, the biphasic insulin secretion profile was defined and the relationship between insulin and glucose concentrations as a possible mechanism of the disease was suggested. Since the study by Bergman et al., mathematical models describing the interaction of insulin and glucose have been extensively studied (see [2] for a review).

**Fig. 1:**
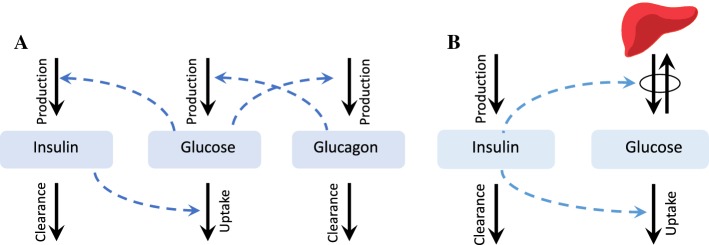
**a** The interaction of glucose, insulin, and glucagon in the body as assumed in this study. **b** The Bergman minimal model [1]. The black arrows represent the processes of production, uptake, and clearance. The blue arrows show the stimulatory or inhibitory effect of glucose, insulin, and glucagon on each of these processes (Color figure online)

A recent experimental study [3], based on a glucagon challenge test, has yielded numerical data about the impact of glucagon on levels of glucose. In this paper a model for this challenge test is proposed. The model captures the interaction of glucose, insulin, and glucagon, and is built upon classical models of glucose–insulin interaction. Figure 1 gives a schematic illustration of this model, together with the Bergman minimal model.

Below, a of list of some distinguishing features of the extended model is presented.

- The Bergman minimal model has two turnover equations, one for insulin and one for glucose, whereas in the model proposed here a third equation for the glucagon concentration in the blood has been added. The Bergman model treats hepatic glucose production (HGP) as a constant independent of the concentration of insulin and glucagon in the blood. In this study, the dependence of HGP on the glucagon concentration needs to be taken into account in order to model the glucagon challenge test.
- The second important feature of the model in the present study is the incorporation of the dynamics of the glucagon receptor. This model has much in common with target-mediated drug disposition (TMDD) models [4], but it has some differences too. The receptor internalization and recycling events are generally not present in TMDD models. Incorporating these events into the model is necessary to explain and justify the results of the challenge test.
- The third difference between the model in the present study and most of the models derived from the minimal model are the strict conditions posed by the setup of the challenge test imposes on the model.
- A fourth feature of the challenge test is that the blood insulin concentration is kept constant during the test. Hence the effect of insulin on glucose production by the liver is neglected in our model.

Many published physiological models of glucose homeostasis do not include the effect of glucagon on the dynamics of glucose and merely focus on the impact of insulin. Here some of the recent studies in which the effect of glucagon, as well as insulin, are included in a model are briefly mentioned. The glucagon-extended minimal model in [5] is built upon the classical Bergman minimal model [1]. Since the model is phenomenological, and the variables do not include a hepatic glucagon concentration, it is not clear how one can add the dynamics of the glucagon receptor to this model. Liu and Tang [6] have incorporated both glycogen and glucagon into a detailed molecular model; for our purposes, however, this model is too detailed and has too many unknown parameters. Even more detailed whole-body models for glucose–insulin–glucagon interaction are given in [7]

Schneck et al. [8] presented a mechanistic model for glucose–insulin–glucagon interaction and used it for studying an oral glucokinase activator. Their model is mostly concerned with modeling the glucagon secretion process whereas the purpose of this study is to model the effect of glucagon on HGP. Schneck et al. captured the effect of glucagon on HGP by using a simple heuristic power expression in which HGP is proportional to (Glucagon)$^{0.79}.$ One might be able to use this simple expression to fit the data of the glucagon challenge test, but this will not yield information on how receptor kinetics affects the HGP.

Peng et al. [9] proposed a detailed model for the glucagon challenge test intended to study the effect of a glucagon receptor antagonist drug on glucose homeostasis in healthy and T2DM subjects. Their focus was to incorporate the PK characteristics of the drug into the model. Another aspect of their model is that the stimulatory effect of glucagon on glucose production is expressed in terms of a simple power expression, which states that HGP is proportional to (Glucagon)$^{4.05}.$ Once again, with this choice of modeling one cannot find out how receptor kinetics affects the HGP.

The glucose–insulin–glucagon pharmacodynamic model by Wendt et al. [10] is concerned with how glucose production varies as a function of glucagon concentration. The crucial modeling choice is the term that describes the dependence of glucose production due to glycogenolysis on the concentration of glucagon in the blood. Wendt et al. use a Michaelis–Menten type model to describe that process. In the present study, similar (Hill-type) kinetics has been used for the same purpose, however not as a function of the plasma concentration of glucagon but of the amount of glucagon-bound receptor on the hepatocyte membrane.

We use data of the glucagon challenge test that was performed on eight subjects at the Center for Human Drug Research in Leiden [11]. For each subject, two data sets are available: one before treatment with the drug and one after 6 weeks of treatment. The parameters of the model are estimated by applying the smooth profiling method, see Supplemental Material, proposed by Ramsay et al. [12] to the data. The parameter estimation was performed for each subject separately. The estimated parameters are further analyzed to determine the efficacy of the drug.

## Methods

### The glucagon challenge test

**Fig. 2:**
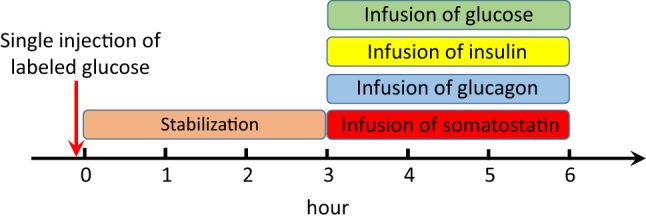
The timeline of the events during the glucagon challenge test is shown. Just before starting the experiment, labeled glucose is injected. The first 3 h is intended for stabilization and measurement of the baselines. In the second 3 h, the infusion of somatostatin stops the endogenous insulin and glucagon secretion by the pancreas. Insulin infusion is adjusted to maintain the baseline concentration, and glucagon infusion is aimed to increase the baseline concentration 2–3-fold

The glucagon challenge test has been proposed as a standard pharmacodynamic tool in pharmacological research related to glucose homeostasis and metabolism [11]. The test takes 6 h: the first 3 h are aimed at stabilising and measuring the baseline concentrations. In the second 3 h of the test, the endogenous pancreatic secretion of insulin and glucagon are inhibited by somatostatin^1^ infusion. Simultaneously, the exogenous infusion of insulin and glucagon are adjusted so that the concentration of insulin in the blood remains constant and the concentration of glucagon in the blood rapidly increases 2–3-fold relative to physiological levels (see Fig. 2).

The data set used in this paper is taken from the glucagon challenge test performed on eight healthy subjects [3]. For each subject, the data set include the following

- The measurement of the concentration of blood glucose, insulin, and glucagon at times 135, 150, 165, 179, 195, 210, 225, 240, 255, 270, 285, 300, 315, 330, 345, and 360 min.
- The HGP rate $F_{hgp}$ and the blood glucose clearance rate *Rd*,  both calculated for the concentration of isotope-enriched glucose [11].

### Mathematical model

In this study it is required that the model describes both periods of the challenge test and satisfies the following conditions:

1. The model should include the effect of glucagon on the glucose dynamics.
2. The model should contain the dynamics of the glucagon receptor so that it becomes possible to study the effect of the internalization and also the effect of the change in the total concentration of the glucagon receptors due to the action of the drug.
3. During the first 3 h of the test, the model should possess stable steady states for all the variables.
4. During the second 3 h the model should be able to fit the data of all subjects with reasonable accuracy.

**Fig. 3:**
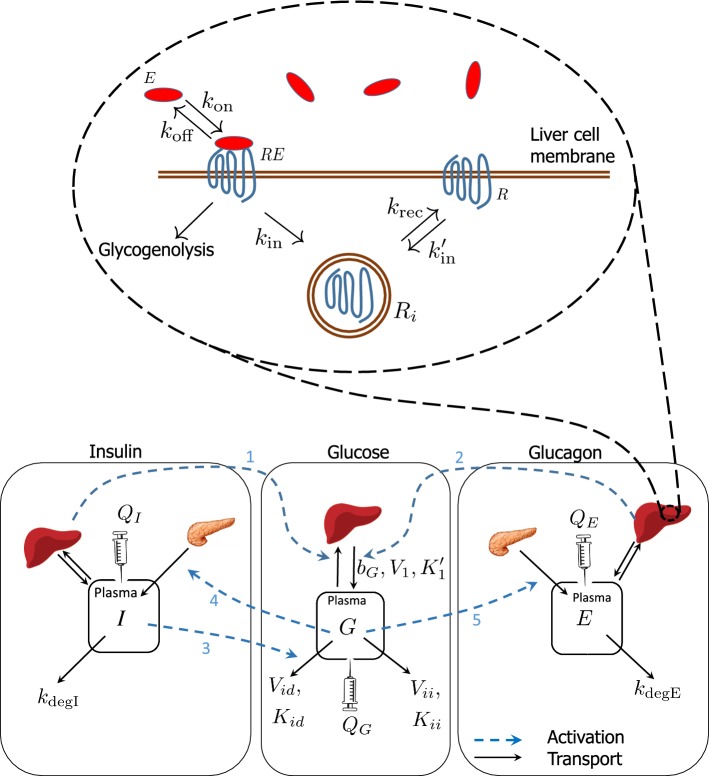
The compartmental model for glucose homeostasis by insulin and glucagon is shown below. The black arrows indicate transport of substances and the blue arrows show how different processes are activated. Insulin and glucagon are both secreted by the pancreas into plasma. A small amount of these two hormones are then extracted from the plasma by the liver. Insulin enhances glucose storage in the liver (arrow 1) and uptake in tissues (arrow 3). Glucagon activates the release of glucose from the liver (arrow 2). Through a feedback mechanism, glucose affects secretion rate of insulin (arrow 4) and glucagon (arrow 5). The glucagon molecules that are extracted by the liver, participate in a set of biochemical reactions that involve the glucagon receptors on the membrane of the liver cells. The description of the parameters is given in Tables 1 and 2 (Color figure online)

The model involves three compartments, one for glucose, one for insulin, and one for glucagon in the blood, with concentrations *G* (g/L), *I* (mU/L), and *E* (pmol/L). The equation for *G* is coupled to a model for the dynamics of the glucagon receptors in a representative liver cell which involves three concentrations $R,\, RE,$ and $R_{i},$ for the three forms of the glucagon receptor: free receptor, glucagon-bound receptor, and the internalized receptor. The whole model is depicted in Fig. 3. All the parameters of the model and their description are presented in Tables 1 and 2.

**Table 1:** The parameters that are fitted to the data

Parameter description	Symbol	Unit
Baseline HGP rate	$b_{G}$	(g/h/L)
Maximal glucagon-dependent HGP rate	$V_{1}$	(1/h)
Apparent dissociation const.	$K'_{1}$	(1)
Maximal *I*-independent *G* consumption	$V_{ii}$	(1/h)
Maximal *I*-dependent *G* consumption	$V_{id}$	(1/h)
MM const. for *I*-independent *G* consumption	$K_{id}$	(g/L)
Degradation rate of insulin	$k_{{\text {degI}}}$	(1/h)
Degradation rate of glucagon	$k_{{\text {degE}}}$	(1/h)
Internalization rate of glucagon-bound receptor	$k_{{\text {in}}}$	(1/h)
Apparent volume of the hepatic interstitial space	$V_{h}$	(L)

**Table 2:** Parameters that are not fitted to the data

Parameter description	Symbol	Unit	Value	
			0–3 h	3–6 h
Infusion rate of glucose	$Q_{G}$	g/h	0	0.24
Infusion rate of insulin	$Q_{I}$	mU/h	0	480
Infusion rate of glucagon	$Q_{E}$	pmol/h	0	4134.7
Distribution volume of glucose	$V_{G}$	L	$\dagger$	4.44
Distribution volume of insulin	$V_{I}$	L	$\dagger$	1.52
Distribution volume of glucagon	$V_{E}$	L	$\dagger$	9.6
Association rate of *E* and *R*	$k_{{\text {on}}}$	1/pmol/h	$3.6\times 10^{-3}$	
Dissociation rate of *E* and *R*	$k_{{\text {off}}}$	1/h	14.4	
Recycling rate of glucagon receptor	$k_{{\text {rec}}}$	1/h	0.18	
MM const. for *I*-independent	$K_{ii}$	g/L	0	
*G* consumption				
Association rate of free receptor	$k'_{{\text {in}}}$	1/h	0	
Total amount of glucagon receptor	$R^{{\text {tot}}}$	pmol	$\star$	
Apparent dissociation const.	$K_{1}$	pmol	$\star$	
Baseline insulin secretion rate	${\overline{F}}_{I}$	mU/h/L	$\ddagger$	
Baseline glucagon secretion rate	${\overline{F}}_{E}$	pmol/h/L	$\ddagger$	

($\dagger$) Volumes of distribution are not defined for the first period since there is no infusion. ($\star$) These parameters are unidentifiable. Only their ratio denoted by $K'_{1}$ is identifiable. ($\ddagger$) These two rates are not present in the second 3 h of the test and not needed in our analysis, hence they are not fixed or fitted

### Insulin compartment

The rate of change of the insulin concentration in the blood is assumed to be due to infusion, pancreatic secretion, and clearance from the blood. Therefore the equation for the rate of change in the concentration of insulin in the blood is assumed to be

1 $$\dfrac{{{\text {d}}} I}{{\text {d}t}} = \underbrace{\dfrac{Q_{I}(t)}{V_{I}}}_{\text {infusion}} + \underbrace{F_{I}}_{\begin{array}{c} {\text {endogenous }}\\ \text {secretion} \end{array}} -\underbrace{k_{{\text {degI}}} I}_{\text {clearance}}, \quad I(0)={\overline{I}}.$$

The first two terms describe the flow of insulin into the compartment through infusion and pancreatic secretion. The last term models the flow of insulin out of the compartment through clearance from the blood. The initial condition at $t=0$ is the baseline insulin level ${\overline{I}}.$ Secretion of insulin in the pancreas is triggered by a high level of glucose in the blood. The secretion rate function $F_{I}(t)$ in Eq. (1) should depend on blood glucose concentration. However, in the setup of the glucagon challenge test, it is assumed that in the first 3 h the secretion rate is constant and in the second 3 h it is equal to zero. Therefore, here this rate does not depend on the glucose concentration, and

$$F_{I}(t) = \left\{ \begin{array}{ll} {\overline{F}}_{I}&{}\quad 0<t<3, \\ 0 &{}\quad 3\le t<6, \end{array}\right.$$

where ${\overline{F}}_{I}$ is the basal secretion rate.

The clearance of insulin is assumed to be a first-order process with rate constant $k_{{\text {degI}}}.$ Finally, $Q_{I}$ is the rate of infusion of insulin into the blood,

$$Q_{I}(t) = \left\{ \begin{array}{ll} 0 &{}\quad 0<t<3, \\ {\overline{Q}}_{I} &{}\quad 3\le t<6, \end{array}\right.$$

and $V_{I}$ is the volume of distribution of insulin.

### Glucagon compartment

Like insulin, the rate of change of the glucagon concentration in the blood is assumed to be due to infusion, pancreatic secretion, and clearance from the blood. Thus the turnover equation is given by

2 $$\dfrac{{{\text {d}}} E}{{\text {d} t}} =\dfrac{Q_{E}(t)}{V_{E}} + F_{E} - k_{{\text {degE}}} E, \quad E(0)={\overline{E}}.$$

The glucagon secretion rate function $F_{E}(t)$ is assumed to be

$$F_{E}(t) = \left\{ \begin{array}{ll} {\overline{F}}_{E} &{}\quad 0<t<3, \\ 0 &{}\quad 3\le t<6, \end{array}\right.$$

which is independent of *G*. It is assumed that glucagon is degraded through a first-order process with the rate constant $k_{{\text {degE}}}$ and injected into the bloodstream at a rate

$$Q_{E}(t) = \left\{ \begin{array}{ll} 0 &{}\quad 0<t<3, \\ {\overline{Q}}_{E} &{}\quad 3\le t<6. \end{array}\right.$$

Here $V_{E}$ is the volume of distribution of glucagon. Note that the data of the glucagon challenge test say little about the dependence of the secretion rate of insulin and glucagon on the glucose concentration because these pancreatic secretions are effectively inhibited by somatostatin. It is only possible to estimate the basal value of these secretion rates, i.e., ${\overline{F}}_{I}$ and ${\overline{F}}_{E}.$

### Glucose compartment

The changes in the concentration of blood glucose are due to its production in the body and its clearance from the blood. During the glucagon challenge test, the only source of glucose production in the body is the HGP. The hepatic glucose production rate $F_{hgp}$ will be discussed in connection with the glucagon receptor dynamics.

During the challenge test, there is a small amount of intravenous glucose infusion at a rate

$$Q_{G}(t) = \left\{ \begin{array}{ll} 0 &{}\quad 0<t<3, \\ {\overline{Q}}_{G} &{}\quad 3\le t<6. \end{array}\right.$$

Glucose is removed from the blood by two types of processes, one insulin-dependent and one insulin-independent. The uptake of glucose into tissues is an insulin-dependent process, whereas brain consumption and renal excretion are examples of insulin-independent processes. The data from the glucagon challenge test show that the total clearance rate (the sum of the two) is affected very little as the glucose level rises.^2^ In view of this observation, Michaelis–Menten type expressions are used for both terms to allow for sub-linear rates. The equation for the time course of glucose concentration in the blood is therefore

3 $$\dfrac{{\text {d}}G}{{\text {d} t}} = \dfrac{Q_{G}(t)}{V_{G}} + F_{hgp} - \dfrac{V_{ii}G}{K_{ii}+G} - \dfrac{V_{id} I \cdot G}{K_{id}+G},$$

with $V_{G}$ being the volume of distribution of glucose, $V_{ii}$ and $V_{id}$ the maximum insulin-independent (*ii*) and insulin-dependent (*id*) uptake rates, and $K_{ii}$ and $K_{id}$ the corresponding Michaelis–Menten constants.

### Some comments on the modeling choices

The design of the glucagon challenge test aims at full control over the concentration of hormones in the blood. To that end, the endogenous secretion of hormones is almost completely inhibited by injecting somatostatin during the second 3 h of the test, and the hormone levels are maintained by infusion instead.

Because of this setup, during the second part of the experiment a one-way relationship between hormones and glucose is assumed: hormone concentrations affect glucose dynamics but not vice versa. A mathematical consequence of this assumption is that the equations for glucagon and insulin concentrations become exactly solvable; the solutions are exponential functions.

Somatostatin takes a short time, 3–4 min, to take full effect. A more realistic assumption would, therefore, be to consider the dependence of these secretion rate functions ($F_{I}$ and $F_{E}$) on glucose during this short section of the second period. This would require additional modeling of the injection of somatostatin and of the interaction between somatostatin and the secretion processes. However, the data (taken every 15 min) have insufficient information about this short period (3–4 min). This means that the corresponding parameters would not be identifiable from the data. For this reason, it was assumed that endogenous hormone secretion instantaneously drops to zero.

### Dynamics of the glucagon receptor

Glucagon receptors on the surface of the hepatocyte membrane trigger a cAMP-mediated pathway that transforms and releases the stored liver glycogen as glucose into the blood. It has been shown that glucagon receptors internalize after stimulation with glucagon [13]. If the stimulation is acute (around 30 min), then the internalized receptors are rapidly recycled back to the surface of the liver cells. However, after chronic stimulation (around 5 h), the internalized receptors are degraded.

G-protein-coupled receptors (GPCRs) undergo desensitization and consequent internalization to ensure proper spatiotemporal regulation of signal transduction in case acute or chronic stimulation occurs [14]. Depending on the pathway two types of desensitization are recognized; *homologous*, when the specific ligand of the receptor is responsible for the decrease in responsiveness, and *heterologous*, when the desensitization pathway is independent of the specific ligand. The glucagon receptor internalizes in both ways [15].

**Fig. 4:**
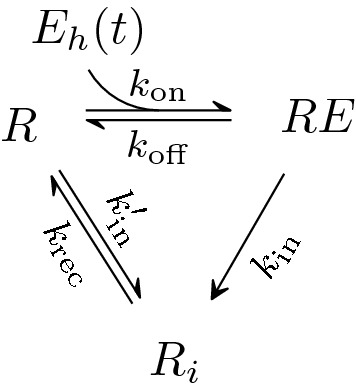
Reaction diagram for receptor binding, internalization, and recycling of glucagon receptor. Variations of this diagram have extensively been used to model the dynamics of other receptors [19]

Given the limited information available about the behavior of the glucagon receptor, all the processes are simplified into some basic reactions presented in Fig. 4. The reactions include the binding of glucagon to its receptor, the internalization of both glucagon receptor *R* and glucagon-bound receptor *RE*,  and recycling of the internalized glucagon receptor $R_{i}.$

The dynamics of the glucagon receptor has only recently been considered in mathematical models of glucose homeostasis. Liu and Tang [6] incorporated a phenomenological model of receptor dynamics into a physiological model for glucose homeostasis. Their model for the glucagon receptor compartment is a heuristic simplification of a more extended model for insulin receptor presented in [16]. Sumner et al. [17] presented a model for liver glucose homeostasis. They assumed a simple GPCR model for the receptor compartment. Their model does not cover the receptor internalization and recycling events. In a study by Schaller et al. [18] a whole-body model for the glucose–insulin–glucagon interaction is presented. The glucagon receptor is modeled as a simple GPCR with internalization and recycling. Apart from the fact that the model by Schaller et al. does not possess a state variable for the glucagon-bound receptor on the membrane of the liver cells, the model developed in the present study is very similar to the one presented in [18, p. 40].

In comparison to the various models described above, the model for the dynamics of the glucagon receptor that is presented here is much simpler, and has the benefit of allowing us to fit parameters from the challenge test data.

### Modeling the receptor dynamics

The reactions involved in modeling the receptor dynamics are summarized in Fig. 4. Glucagon in the liver binds to the glucagon receptor on the surface of the hepatocyte membrane. We assume that this binding happens at a rate that is proportional to both the amount of the glucagon molecules in the hepatic interstitial space, denoted by $E_{h},$ and the amount of surface receptors *R*. Then the glucagon-bound receptor, *RE*,  dissociates and internalizes via two first-order processes with rate constants $k_{{\text {off}}}$ and $k_{{\text {in}}}$ respectively. The internalized receptors $R_{i}$ recycle back to the surface of the hepatocyte membrane with the rate constant $k_{{\text {rec}}}.$ The free receptors also internalize at a rate $k'_{{\text {in}}} R.$

Besides the reaction rates, the following modeling assumptions are made:

1. The concentration of glucagon in the hepatic interstitial space, i.e., around glucagon receptors, is instantaneously equilibrated with the concentration of glucagon in the blood. We thus assume that
  $$E_{h}(t) = V_{h} \cdot E(t),$$
  where $V_{h}$ can be regarded as the volume of the hepatic interstitial space. It is assumed to be a different constant for every subject, and it will be estimated later. Note that $V_{h}$ has the units of volume (L), since *E* has the unit pmol/L and $E_{h},$ the total amount of glucagon molecules in the hepatic interstitial space, has units of pmol.
2. It is assumed that the binding of glucagon molecules to the glucagon receptor does not affect the concentration of glucagon in the blood, assuming that only a small fraction of the glucagon molecules binds to the receptors. Therefore in the model of the receptor [Eq. (4)], the concentration of glucagon $E_{h}(t)$ acts only as a time-dependent input function, and the dynamics of the glucagon compartment does not depend on the receptor dynamics.
3. Another assumption is that the synthesis rate of new receptors is equal to the degradation rate. Therefore the conservation of the total number of receptors is enforced, i.e., $R+RE+R_{i}=R^{{\text {tot}}}.$

The equations for the dynamics of the concentrations of the three forms of the glucagon receptor are

4 $$\begin{aligned}\dfrac{{{\text {d}}} R}{{\text {d}} t}&= -k_{{\text {on}}} V_{h} \cdot E(t)\cdot R + k_{{\text {off}}} RE -k'_{{\text {in}}} R + k_{{\text {rec}}} R_{i}, \\ \dfrac{{\text {d}} RE}{{\text {d}} t}&= + k_{{\text {on}}} V_{h} \cdot E(t)\cdot R - k_{{\text {off}}} RE -k_{{\text {in}}} RE, \\ \dfrac{{{\text {d}}} R_{i}}{{{\text {d}}} t}&=+ k_{{\text {in}}} RE +k'_{{\text {in}}} R - k_{{\text {rec}}} R_{i}. \\ \end{aligned}$$

Conservation of the total amount of receptors allows to describe the receptor dynamics with only two differential equations, one for *R* and one for *RE*. For reasons of parameter identifiability, the concentrations of $R,\,RE,$ and $R_{i}$ are scaled with $R^{{\text {tot}}}$ and introduce the dimensionless variables

$$r=\dfrac{R}{R^{{\text {tot}}}}, \quad r_{e} = \dfrac{RE}{R^{{\text {tot}}}},\quad r_{i} = \dfrac{R_{i}}{R^{{\text {tot}}}}.$$

After scaling and using the conservation law to elimate $r_{i},$ the equations for *r* and $r_{e}$ become

5 $$\begin{aligned} \dfrac{{\text {d}} r}{{\text {d}} t}&= -k_{{\text {on}}} V_{h} \cdot E(t)\cdot r + k_{{\text {off}}} r_{e} -k'_{{\text {in}}} r + k_{{\text {rec}}} (1-r-r_{e}), \\ \dfrac{{\text {d}} r_{e}}{{{\text {d}}} t}&= + k_{{\text {on}}} V_{h} \cdot E(t)\cdot r - k_{{\text {off}}} r_{e} -k_{{\text {in}}} r_{e}. \end{aligned}$$

For a given constant concentration of glucagon, ${\overline{E}},$ the receptor compartment always possesses a unique attracting steady state $({\overline{r}},{\overline{r}}_{e}),$

6 $$\begin{aligned} {\overline{r}}&= \dfrac{\left( k_{{\text {off}}}+k_{{\text{ in }}}\right) k_{{\text {rec}}}}{(k'_{{\text {in}}}+k_{{\text {rec}}})(k_{{\text {off}}}+k_{{\text {in}}})+( k_{{\text {in}}}+k_{{\text {rec}}})k_{{\text {on}}} V_{h} {\overline{E}} }, \\ {\overline{r}}_{e}&= \dfrac{k_{{\text {rec}}}k_{{\text {on}}} V_{h} {\overline{E}} }{(k'_{{\text {in}}}+k_{{\text {rec}}})(k_{{\text {off}}}+k_{{\text {in}}})+( k_{{\text {in}}}+k_{{\text {rec}}})k_{{\text {on}}} V_{h} {\overline{E}} }. \end{aligned}$$

### The hepatic glucose production rate

Liver glucose production is known to be activated by glucagon and inhibited by insulin. In the setup of the glucagon challenge test, the concentration of insulin in the blood is kept close to constant throughout the test. Therefore it is not possible to study the dependence of $F_{hgp}$ on the blood insulin concentration. However, $F_{hgp}$ should be a function of the glucagon-bound receptor, *RE*,  since this species is the one that activates the cAMP-mediated pathway of glycogenolysis. In fact, for GPCRs, there exists a variety of empirical and mechanistic models that relate the ‘rate of response’ to the concentration of agonist-bound receptor [20]. Three different possibilities were investigated for this rate:

- A linear dependence, $$b_{G}+V_{1} RE,$$
- A Michaelis–Menten-type expression $b_{G}+\dfrac{V_{1} RE}{K_{1}+RE},$
- A Hill-type expression of the form $b_{G}+\dfrac{V_{1} (RE)^{n}}{K_{1}^{n}+(RE)^{n}},$

where $b_{G}$ is a constant that will be fitted. Among these three rates, the Hill-type expression gave the best fitting to the data. The Hill coefficient *n* is fixed to be equal to 2. The reason it was not estimated was that by not fixing it the model would become very nonlinear with respect to the parameter. That would be problematic because the parameter estimation method that is chosen in this study works best for systems that are linear in their parameters. Rewriting the expression for $F_{hgp}$ in terms of the rescaled concentration $r_{e}$ gives

$$F_{hgp} = b_{G}+\dfrac{V_{1} r_{e}^{2}}{{K'}_{1}^{2}+r_{e}^{2}}, \quad K'_{1} = K_{1}/R^{{\text {tot}}}.$$

### The full model

Recall that there are two forms of the above equations during the challenge test: the first 3 h of steady state without any infusion

7a $$\begin{aligned} 0&= b_{G}+\frac{V_{1} \overline{r_{e}}^{2}}{{K'}_{1}^{2}+\overline{r_{e}}^{2}} - \frac{V_{ii}{\overline{G}}}{K_{ii}+{\overline{G}}} \\&\quad - \frac{V_{id} {\overline{I}} {\overline{G}}}{K_{id}+{\overline{G}}}, \end{aligned}$$

7b $$0= {\overline{F}}_{I} - k_{{\text {degI}}} {\overline{I}},$$

7c $$0= {\overline{F}}_{E}- k_{{\text {degE}}} {\overline{E}},$$

7d $${\overline{r}}= \dfrac{1}{(k'_{{\text {in}}}+k_{{\text {rec}}}) \dfrac{k_{{\text {in}}}+k_{{\text {rec}}}}{k_{{\text {off}}}+k_{{\text {in}}}} k_{{\text {on}}} V_{h} {\overline{E}} },$$

7e $${\overline{r}}_{e}= \dfrac{k_{{\text {on}}} V_{h} {\overline{E}} }{ (k_{{\text {off}}}+k_{{\text {in}}}) }{\overline{r}},$$

and the second 3 h with infusion and clamped hormone secretion.

8a $$\begin{aligned} \dfrac{{{\text {d} }}G}{{\text {d} t}}&= \dfrac{Q_{G}}{V_{G}} + b_{G}+\dfrac{V_{1} r_{e}^{2}}{{K'}_{1}^{2}+r_{e}^{2}} - \dfrac{V_{ii}G}{K_{ii}+G} \\&\quad - \dfrac{V_{id} I G}{K_{id}+G}, \quad G(3)={\overline{G}}, \end{aligned}$$

8b $$\dfrac{{{\text {d}}} I}{{\text {d} t}}= \dfrac{Q_{I}}{V_{I}} - k_{{\text {degI}}} I, \quad I(3)={\overline{I}},$$

8c $$\dfrac{{\text {d}} E}{{\text {d} t}}=\dfrac{Q_{E}}{V_{E}} - k_{{\text {degE}}} E, \quad E(3)={\overline{E}},$$

8d $$\begin{aligned} \dfrac{{{\text {d}}} r}{{{\text {d}}} t}&= -k_{{\text {on}}} V_{h} \cdot E\cdot r \\&\quad + k_{{\text {off}}} r_{e} -k'_{{\text {in}}} r + k_{{\text {rec}}} (1-r-r_{e}), \quad r(3)={\overline{r}}, \end{aligned}$$

8e $$\dfrac{{{\text {d}}} r_{e}}{{{\text {d}}} t}= + k_{{\text {on}}} V_{h} \cdot E\cdot r - k_{{\text {off}}} r_{e} -k_{{\text {in}}} r_{e}, \quad r_{e}(3)={\overline{r}}_{e}.$$

The steady-state equations yields relations between the parameters and the basal concentrations ${\overline{G}},\,{\overline{I}},\, {\overline{E}},\,{\overline{r}},$ and ${\overline{r}}_{e}.$ The basal concentrations are fit to the data of 0 to 3 h. Therefore five relations between the parameters are obtained. When fitting the data for the second 3 h to the ODEs, we require that these five relations continue to be satisfied. The setup of the challenge test is such that these basal concentrations serve as initial conditions for the differential equations (8).

Another requirement for fitting the data is also imposed. The basal HGP rate, denoted by ${\overline{F}}_{hgp},$ is obtained by averaging the measured amount of $F_{hgp}$ for the first 3 h of the test. Then, it is naturally required, due to Eq. (7a) and the definition of $F_{hgp},$ that at the steady state phase, the production terms and the clearance terms in the glucose equation satisfy

9 $${\overline{F}}_{hgp} =b_{G}+\dfrac{V_{1} \overline{r_{e}}^{2}}{{K'_{1}}^{2}+\overline{r_{e}}^{2}} = \dfrac{V_{ii}{\overline{G}}}{K_{ii}+{\overline{G}}} + \dfrac{V_{id} {\overline{I}} {\overline{G}}}{K_{id}+{\overline{G}}}.$$

### Model fitting and parameter estimation

During the glucagon challenge test, it is impossible to directly measure concentrations of substances in the liver. Nevertheless, it is desirable to obtain an estimate for such concentrations by combining the mathematical modeling with the data from the blood concentrations of glucose, insulin, and glucagon. The data fitting is challenging due to the presence of unmeasured variables in the set of differential equations.

Due to parameter identifiability issues that are discussed in Supplemental Material, it is not possible in our setting to estimate the total number of glucagon receptors from the current model and data. However, what can be estimated is $K'_{1} = K_{1}/R^{{\text {tot}}}.$ In [6] and later studies the total amount of the glucagon receptors is taken to be equal to the total amount of the insulin receptors reported in [16] and it is about 1 pmol. This estimate of the total number of glucagon receptors validates our assumption that the concentration of glucagon in blood, which is about 14.3 pmol/L, does not change much when glucagon binds to its receptor on the surface of the liver cells.

To address the issues of parameter identifiability and to help the parameter fitting routine, some of the parameters are set to be constants reported in previous studies.

The glucagon receptor is a GPCR. The experimental values of $k_{{\text {on}}}$ for 10 different GPCRs are gathered in [21] which shows that most measurements report a value between $6\times 10^{-5}$ to 0.06 (pM h)$^{-1}.$ The value that was used, $k_{{\text {on}}}= 0.0036$ (pM h)$^{-1}$ is in the middle of this range and was taken from [22]. The dissociation rate constant $k_{{\text {off}}} = 0.24$ min$^{-1}$ was taken from the same reference. Therefore the equilibrium dissociation constant is $K_{d} = 4000$ pmol.

It is assumed that the recycling rate of glucagon receptors is equal to the recycling rate of the insulin receptor $k_{{\text {rec}}}=0.003$ min$^{-1}$ as reported in [16]. In [18, p. 120] this value is obtained by fitting and is reported to be 0.01 min$^{-1}.$ The former value will be used for the fitting in this paper.

To estimate some of the parameters in the set of differential equations (8) the smoothing approach proposed by Ramsay et al. [12] is used. For the three parameters $k_{{\text {on}}},\,k_{{\text {off}}},$ and $k_{{\text {rec}}}$ the literature values were used as discussed, and were kept equal to the same constant for all subjects. During the estimation procedure, two of the parameters, $k'_{in}$ and $K_{ii},$ collapsed to zero. Therefore they were set to be zero for all subjects. The infusion rates and the volumes of distribution are also equal for all subjects and presented in Table 3.

**Table 3:** The values of infusion rates and the volumes of distribution obtained from [23]

${\overline{Q}}_{G}$	${\overline{Q}}_{I}$	${\overline{Q}}_{E}$	$V_{G}$	$V_{I}$	$V_{E}$
0.24 g/h	480 mU/h	4134.7 pmol/h	4.44 L	1.52 L	19.6 L

**Fig. 5:**
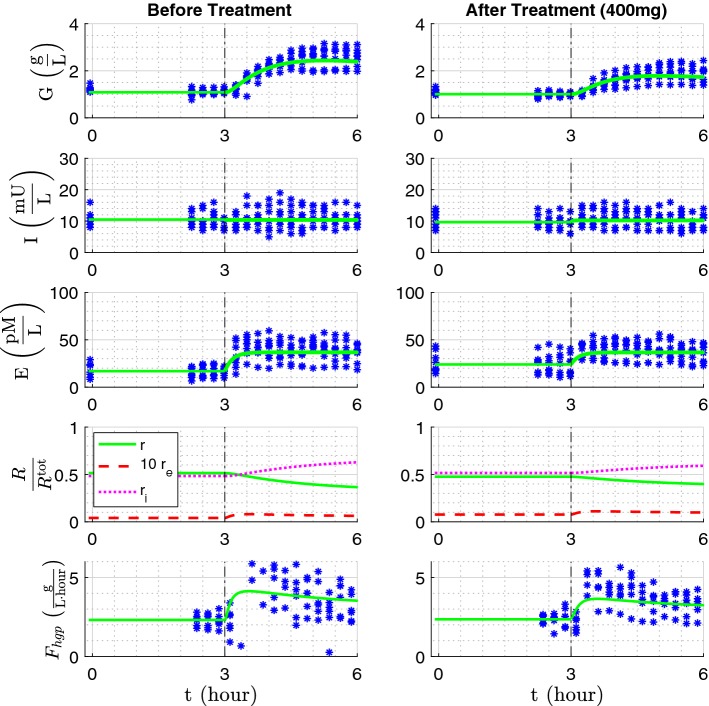
The plot of all the data points and the solution to the model equations using average parameters reported in Table 4, before and after treatment

For every subject, the parameter estimation was performed twice: once with the data obtained before treatment, and once with the data obtained after treatment. During the parameter estimation, it was required that the steady state equations as well as the relation (9) are satisfied. The details of the estimation method are explained in Supplemental Material, and the results of parameter estimation are presented in Table 4. A plot of the solution of the ODEs using the average parameters is shown in Fig. 5 and the individual fittings are depicted in Supplemental Material.

**Table 4:** The average and the standard deviation of estimated parameters, p-values obtained from comparing the before and after treatment parameters using the Wilcoxon signed rank test together with the Benjamini–Hochberg FDR are reported. The parameter $G_{max}$ is directly calculated from the data. A complete table with the fitted parameters for every subject is provided in the Supplemental Material

	Unit	Before average	Before SD	After average	After SD	p-values	FDR
$V_{1}$	(1/h)	5.63	1.97	6.81	1.85	0.0391	0.143
$V_{ii}$	(1/h)	0.896	0.319	0.936	0.294	0.742	1
$V_{h}$	(L)	4.65	2.28	4.96	1.77	1	1
$K_{id}$	(g/L)	11	6.62	10.3	5.5	0.945	1
$k_{{\text {degI}}}$	(1/h)	30.5	8.27	30.9	7.93	0.641	1
$k_{{\text {degE}}}$	(1/h)	5.73	1.84	5.76	1.51	0.844	1
$k_{in}$	(1/h)	21.5	15.2	12.1	3.15	0.109	0.301
$K'_{1}$	–	0.00501	0.00257	0.0109	0.0045	0.00781	0.043
$b_{G}$	(g/L/h)	0.0804	0.228	0.18	0.371	0.945	1
$V_{id}$	(1/h)	1.52	0.963	1.66	0.768	0.547	1
$G_{{\max }}$	(g/L)	2.71	0.353	2	0.336	0.00781	0.043

### Analysis of the data

The fitted parameters for both before and after treatment are presented in Table 4. To find out which parameters were affected by the treatment, the Wilcoxon signed-rank test was performed on each of the estimated parameters separately. The Wilcoxon signed-rank test is used to compare the distribution of paired observations. The null hypothesis, in this case, is that the difference between the median of the two data sets is zero. We have calculated the *p*-values of the test for each parameter (see Table 4). The *p*-values are corrected for multiple hypothesis testing with the Benjamini–Hochberg false discovery rate (FDR) procedure [24]. Only the ones with an FDR less than 0.05 should be declared significant here which means only for the parameter $K'_{1}$ the *p*-value and the FDR satisfy the condition ($p=0.00781$ and FDR $= 0.043$). As this parameter is inversely proportional to the total number of receptors, we infer that the drug has effectively changed the number of receptors. For other parameters, the null hypothesis can not be rejected. Therefore it cannot be concluded that the drug had an effect on them.

Another measure with which the efficacy of the drug can be studied is the change in the peak value of the measured glucose concentration, see the last row in Table 4. The reduction in $G_{max}$ after treatment is also statistically significant, $p=0.00781$ and FDR $= 0.043.$

Recall that $K'_{1}=K_{1}/R^{{\text {tot}}}$ and assume that $K_{1}$ does not change considerably after treatment with the drug, i.e., $\left( K_{1}\right) _{{\text {before}}} =\left( K_{1}\right) _{{\text {after}}}.$ Then the relative change in the total number of receptors denoted by $\varDelta$ can be calculated as:

$$\begin{aligned} \varDelta&= \dfrac{\left( R^{{\text {tot}}}\right) _{{\text {before}}} -\left( R^{{\text {tot}}}\right) _{{\text {after}}} }{\left( R^{{\text {tot}}}\right) _{{\text {before}}} } \\&= \dfrac{\left( \dfrac{R^{{\text {tot}}}}{K_{1}}\right) _{{\text {before}}} - \left( \dfrac{R^{{\text {tot}}}}{K_{1}}\right) _{{\text {after}}} }{\left( \dfrac{R^{{\text {tot}}}}{K_{1}}\right) _{{\text {before}}}} \\&=\dfrac{\left( K'_{1}\right) _{{\text {before}}}^{-1} - \left( K'_{1}\right) _{{\text {after}}}^{-1} }{\left( K'_{1}\right) _{{\text {before}}}^{-1} }. \end{aligned}$$

The average value $\varDelta$ is calculated in two different ways. First the value of $\varDelta$ for each subject is calculated separately. In this way, the average value is $AV(\varDelta )=-0.47 \pm 0.25.$

It is also possible to use the average values of $K'_{1}$ from Table 4 and calculate the relative decrease,

$$\begin{aligned}&\dfrac{\left( AV\left[ \left( K'_{1}\right) _{{\text {after}}}\right] \right) ^{-1} - \left( AV\left[ \left( K'_{1}\right) _{{\text {before}}}\right] \right) ^{-1} }{ \left( AV\left[ \left( K'_{1}\right) _{{\text {before}}}\right] \right) ^{-1} }\\&\quad =\dfrac{1/0.011 -1/0.005 }{1/0.005 } \\&\quad = - 0.55. \end{aligned}$$

In this way, a 55% decrease in the total number of glucagon receptors is predicted. Both ways of calculating the relative change in the total number of glucagon receptors show a significant decrease after treatment.

The fitted value of $b_{G}$ for subjects 30, 31 (after treatment), and subject 38 (before treatment) are orders of magnitude higher than the values of this parameter for other subjects. This discrepancy is the result of our choice to fix the Hill coefficient to 2 and compensate for it by adding $b_{G}$ to the HGP rate $F_{hgp}.$ It was observed that by setting the parameter $b_{G}$ to 0 for these two subjects, the fitting does not get close enough to the data. This problem could have been avoided by choosing to estimate the Hill coefficient, however that would make our model too nonlinear and the parameter fitting algorithm would fail to fit the data.

The time scales of the receptor kinetics are very long and it does not reach a steady state for most of the subjects. To explain this outcome, note that this speed is dictated by the rates $k_{{\text {on}}} V_{h} E \approx 0.0036\,*\,4.65\,*\,20=0.335$ (1/h), $k_{{\text {off}}} = 14.4$ (1/h), $k_{{\text {rec}}} = 0.18$ (1/h), and $k_{{\text {in}}} 12.1$ (1/h). After infusion of glucagon, the rate limiting step that prevents stabilization of the receptor subsystem is the binding step of glucagon to its receptor. The value of $k_{{\text {on}}}$ was taken from literature and, as stated previously, is consistent with the experimentally measured values of $k_{{\text {on}}}$ for other GPCRs. The parameter fitting procedure has the freedom to choose a bigger value for $V_{h}$ and increase the speed of this rate limiting binding process. But it successfully fits the data at the given rate, resulting in a slow kinetics of the receptor subsystem.

## Discussion

In this paper, we started by describing the interaction between glucose and two associated regulatory hormones: insulin and glucagon. Understanding the mechanisms involved in the complex dynamics of these three will help in designing better ways to treat diabetes. A special focus was on recent data obtained by a glucagon challenge test, which is a standardized way of measuring the effect of glucagon on hepatic glucose release [3]. A mathematical model for glucose–insulin–glucagon interaction was proposed, and used to interpret the clinical trial data obtained from the glucagon challenge test.

The model contains a subsystem for the glucagon receptors on the surface of the liver cells. This enabled us to answer the two questions that were posed in the introduction. The first question was to show how the glucagon receptor internalization leads to tolerance against glucagon-induced hyperglycemia. In Fig. 5, where the average response is depicted, it is observed that in the second 3 h of the test there is a decrease in the amount of free receptor on the surface of the liver cells; around 15% for before treatment data and 8% for after-treatment data, a difference which is significant. Most of these free receptors first bind to the glucagon molecules and then internalize very fast. A small portion remains bound. The time course of the concentration of $r_{e}$ and the value of $F_{hgp}(r_{e})$ show an initial rise due to the abundance of glucagon followed by a slow decay due to internalization. The average values for the internalization rate $k_{{\text {in}}},$ 21.022 1/h for before treatment and 12.05 1/h for after treatment, imply that the internalization process is quite fast.

The second question dealt with the quantitative assessment of the efficacy of a novel antisense drug aimed at blocking the production of the glucagon receptor. By fitting and comparing the data of the glucagon challenge test before and after treatment with the drug (400 mg administration), an average decrease of 47 to 55% was found in the number of glucagon receptors in liver cells.

A few comments on some aspects of this work are presented below.

### The size of the model

We claim that the set of Eqs. (7) and (8) describes the least complex model that is (a) based on the physiology of glucose homeostasis, (b) based on the biochemistry of glucagon receptor, and (c) can successfully fit the data of the glucagon challenge test. By using simpler kinetics for the rates or by modeling with fewer equations, some of the three conditions required for the modeling will get violated.

### The glucagon receptor compartment

Three basic reactions—binding, internalization, and recycling—were introduced to represent the complex systems biology of the glucagon receptor. The model is complex enough to describe the data, yet not too complex to encounter parameter identifiability issues. In case more data on the action of glucagon receptor are available, the model should be improved to include more details such as the binding of glucagon to the GLP-1 receptor [25], or different modes of internalization of the glucagon receptor [15].

### The parameter estimation method

ODE parameter inference is a notoriously difficult computational problem. The smooth profiling method was chosen because compared to various other methods that require numerical solution of ODEs, this method is faster and more successful in avoiding local optima [26]. It can also handle problems with unmeasured components which is necessary for the problem dealt with in this study. In the present study simple application of nonlinear least squares estimation is not possible because the set of equations is not explicitly solvable. The use of NONMEM also brought some limitations to the fitting. Performing parameter estimation on the entire 6 h of the test was problematic due to the very long time it took to stabilize in the initial phase of the test as well as the need to introduce step functions for infusions which slows down the fitting time even further. Alternatively, one could consider using NONMEM only on the 3 h of the challenge, but then glucose baseline needs to be expressed in terms of other parameters while the algebraic steady state equations are not explicitly solvable.

### Tuning parameters of the smooth profiling method

A drawback of the smooth profiling method is that it requires extensive tuning of control parameters *w* and $\lambda$ to gradually update the initial guess, starting with large values for $\lambda ,$ and eventually reach an optimum fit. But once a satisfactory fit is found, the optimum solution is almost independent of perturbations of the tuning parameters. A slight change in the tuning parameters will not result in a different estimate for parameters. These parameters had to be tuned for each separate fit. The method was not used in a mixed effect setting where all data are treated at once. Other approaches, such as a Bayesian approach, could provide a convenient framework for dealing with mixed effect models, and that remains for future work.

### Applicability of this study

The glucagon challenge test together with the model and the data fitting procedure presented in this paper could be used to study the efficacy of any glucagon receptor antisense drug. Moreover, the model presented here, or some extension of it, could be incorporated into recently developed models for an artificial pancreas [27].

## Electronic supplementary material

Below is the link to the electronic supplementary material.

Supplementary material 1 (PDF 665 kb)

## Footnotes

1 Somatostatin is a hormone which inhibits the pancreatic secretion of glucagon and insulin.

2 Van Dongen also makes this observation [11, p. 271].
