# Supplementary material for: Mathematical modeling of the glucagon challenge test
Source: J Pharmacokinet Pharmacodyn. 2019 Sep 30;46(6):553–64. doi: 10.1007/s10928-019-09655-2 (PMC6868112; doi:10.1007/s10928-019-09655-2)
Supplement: Supplementary file 1 — Supplementary material 1 (PDF 665 kb) [file 10928_2019_9655_MOESM1_ESM.pdf]

Mathematical Modelling of the Glucagon Challenge Test

Saeed Masroor · Marloes G.J. van Dongen · Ricardo Alvarez-Jimenez · Koos Burggraaf · Lambertus A. Peletier · Mark A. Peletier

the date of receipt and acceptance should be inserted later

Supplementary material

In the text below we number equations, figures, and tables consecutively to the main text.

Smooth Profiling method

In this paper, we used the parameter estimation method proposed by Ramsay *et al.* [3], and we extensively used the MATLAB functions written by Giles Hooker [1]. Given a set of differential equations for $X \in \mathbb{R}^n$,

$$\frac{\mathrm{d}X}{\mathrm{d}t} = f(X, t, \theta),$$

that depend on a set of parameters represented in a vector θ, we have a set of observations Y_i at times t_ℓ for a subset of variables X_i. The approximation $\hat{X}(t)$ of the state variable $X(t)$ is expressed in the form of an expansion

$$\hat{X}_i(t) = \sum_{k=1}^{K_i} c_{ik}\phi_{ik}(t),$$

S. Masroor
Maastricht University, The Netherlands

M. G.J. van Dongen
Netherlands Cancer Institute, Amsterdam, The Netherlands

R. Alvarez-Jimenez
Amsterdam University Medical Center, Amsterdam, The Netherlands

J. Burggraaf
Centre for Human Drug Research, Leiden, The Netherlands

L. A. Peletier
Leiden University, Leiden, The Netherlands

M. A. Peletier
Eindhoven University of Technology, The Netherlands
E-mail: m.a.peletier@tue.nl

where K_i is the number of basis functions chosen for X_i, and the basis functions ϕ_{ik} are chosen in a manner that they can capture the time variation in the data. The method of Ramsay *et al.* aims to estimate all coefficients c_{ik} and all parameters θ. For that, a two-stage optimization procedure, the so-called inner and the outer optimizations, is performed.

In the inner optimization, the parameters are kept fixed and the coefficients c_{ik} are chosen to minimize the functional

$$G(\hat{X}, \theta, \lambda) := \sum_{i=1}^{n} \{w_i \|Y_i - \hat{X}_i\|^2 + \lambda_i \int \Big(\mathrm{d}\hat{X}/\mathrm{d}t - f(\hat{X}, t, \theta)\Big)^2 \mathrm{d}t\}. \tag{10}$$

The parameters w_i and λ_i allow tuning the degree of fidelity to the data and to the differential equation. The values of these control coefficients that have been used for fitting are presented in Table 6. Once the coefficients c_{ik} are determined from the inner optimization, the parameters θ are varied to minimize

$$J(\theta, \lambda) = \sum_{i=1}^{n} \|Y_i - \hat{X}_i(t_\ell|\theta)\|^2 \tag{11}$$

which measures the distance between the data and the approximation. Whenever θ is changed in the outer optimization, $\hat{X}$ is re-estimated from the inner optimization. Minimization of (11) is performed via a Gauss-Newton procedure.

The fitting starts with a rough initial guess for the parameters. Those elements of the tuning parameter w corresponding to glucose, insulin, and glucagon are chosen to be the inverse of the standard deviation of the last 8 data points.

The tuning parameter λ is chosen to be small at the beginning. The parameters that from result from fitting with a small value of λ are used as an initial guess for a fitting with a larger value of λ. This process is continued until increasing λ does not increase the quality of the fitting. The quality of the fitting is assessed by eye.

Parameter identifiability

Before performing any parameter fitting routine on a mathematical model in terms of ODEs, one needs to make sure that the parameters of the model can be uniquely determined from the available measurements. Parameter identifiability is defined as the capability to uniquely obtain the parameters of a system from input-output measurements. Here we perform such an analysis on the set of equations (8). We note that the equations for I and E are decoupled from the rest. The forms of these two equations are simple and identifiability of parameters follows easily. The parameters k_{degI} and k_{degE} are identifiable and we will estimate them. The two infusion rates Q_I and Q_E are known and we chose to keep the volumes of distribution V_E and V_I the same for all the subjects (see Table 3).

For the rest of the equations, namely the equations for G, r, and r_e, we use the Mathematica package presented in [2] to perform a structural identifiability analysis. The concentrations of glucagon $E(t)$ and insulin, denoted by $Ins(t)$ in the code below, act as input functions to the system. The measured variable is $G(t)$. The initial conditions for r and r_e are symbolically stated in terms of the parameters according to (7). The code and its output are as follows.

```
In[119]:= Clear["Global`"];
Needs["IdentifiabilityAnalysis`"];
deq = {G'[t] == QG / VG + bG + V1 * (re[t])^2 / ((K1 / Rtot)^2 + (re[t])^2)
        - Vii * G[t] / (Kii + G[t]) - Vid * Ins[t] * G[t] / (Kid + G[t]), G[0] == Gss,
    r'[t] == -kon * Vh * E[t] * r[t] + koff * re[t]
        - kpin * r[t] + krec * (1 - r[t] - re[t]),
    r[0] == 1 / ((kin + kpin) + (kin+krec)/(koff+kin) kon * Vh * Ess),
   re'[t] == kon * Vh * E[t] * r[t] - koff * re[t] - kin * re[t],
   re[0] == (kon * Vh * Ess)/(koff + kin) * 1 / ((kin + kpin) + (kin+krec)/(koff+kin) kon * Vh * Ess)};
params = {QG, VG, bG, V1, K1, Rtot, Vii, Kii, Vid,
    Kid, Vh, kon, koff, kpin, krec, kin, Gss, Ess};
iad = IdentifiabilityAnalysis[{deq, G[t]}, {G, r, re}, params, t, {Ins, E}];
iad["IdentifiableQ"]
iad["NonIdentifiableParameters"]
iad["DegreesOfFreedom"]

Out[1]=    False

Out[2]=    {bG, K1, kon, QG, Rtot, VG, Vh}

Out[3]=    4
```

The result states that the model is not identifiable. The 7 unidentifiable parameters are shown with the additional information that there are four degrees of freedom in the unidentifiable parameters. To resolve that issue we note that the three parameters Q_G, V_G, and b_G appear together in only one place. One degree of freedom is for the ratio Q_G/V_G and one other is for the sum $Q_G/V_G + b_G$. Since the values of Q_G and V_G are known, these two degrees of freedom are resolved. The third degree of freedom arises because k_{on} and V_h always appear together in terms of the product $k_{\mathrm{on}}V_h$. If we fix the value of k_{on} this degree of freedom will also be resolved. Finally the last degree of freedom comes from the ratio K_1/R^{tot}. Since we do not have information for the values of these two parameters, we introduced $K_1' = K_1/R^{\mathrm{tot}}$ and we will estimate this scaled parameter instead.

Now we replace K_1/R^{tot} by K_1' and fix the value of k_{on}, Q_G, and V_G and check the identifiability of the system again.

```
In[149]:= Clear["Global`"];
Needs["IdentifiabilityAnalysis`"];
QG = .24; VG = 4.44; kon = 6 * 10^-5;
deq = {G'[t] == .24 / 4.44 + bG + (V1 * (re[t])^2) / ((Kp1)^2 + (re[t])^2)
          - (Vii * G[t]) / (Kii + G[t]) - (Vid * Ins[t] * G[t]) / (Kid + G[t]), G[0] == Gss,
      r'[t] == -kon * Vh * E[t] * r[t] + koff * re[t]
          - kpin * r[t] + krec * (1 - r[t] - re[t]),
      r[0] == 1 / ((kin + kpin) + (kin+krec)/(koff+kin) kon * Vh * Ess),
      re'[t] == kon * Vh * E[t] * r[t] - koff * re[t] - kin * re[t],
      re[0] == (kon * Vh * Ess) / (koff + kin) * 1 / ((kin + kpin) + (kin+krec)/(koff+kin) kon * Vh * Ess)};
params = {bG, V1, Kp1, Vii, Kii, Vid,
    Kid, Vh, koff, kpin, krec, kin, Gss, Ess};
iad = IdentifiabilityAnalysis[{deq, G[t]}, {G, r, re}, params, t, {Ins, E}];
iad["IdentifiableQ"]
iad["NonIdentifiableParameters"]
iad["DegreesOfFreedom"]

Out[1]=   True

Out[2]=   {}

Out[3]=   0
```

The model is now identifiable. However, this means that if there are many measurements available, we can determine the parameters. Since we do not have a lot of measurements, we help the parameter fitting routine by choosing to fix some more parameters of the model.

Fitting results

This section contains the details and the complete results of the fitting for the 8 subjects. In Table 6 the control coefficients λ and w are reported. In Figure 6 we have plotted the average response of the before-treatment data for the 8 subjects studies in this paper against the data from another 8 placebo subjects. In this plot we aim to show the predictive value of model and the fitted parameters. The rest of the figures are individual fittings for the 8 subjects.

Table 6: The values of control coefficients λ and w in the inner optimization problem (10) for each fit. The values of w are chosen to be equal to the inverse of the variance of the last 8 data points.

Subject	λ_1	λ_2	λ_3	λ_4	λ_5	w_1	w_2	w_3	w_4	w_5
30b	1000	10	10	1000	1000	8.1817	1.0916	0.18685	1	1
30a	1000	10	10	100	100	11.4317	0.59161	0.18962	1	1
31b	1000	100	100	1000	1000	3.8315	0.96609	0.24087	1	1
31a	4000	10	10	100	100	8.3493	1.3229	0.20103	1	1
32b	2800	10	10	700	900	4.1943	1.0801	0.23512	1	1
32a	4000	10	10	4000	4000	7.1682	1.8708	0.36156	1	1
33b	1000	10	10	100	100	13.2217	2.1602	0.25556	1	1
33a	2800	10	10	700	900	20.5239	0.68599	0.44136	1	1
35b	4000	10	10	2000	2000	4.7904	0.70711	0.23289	1	1
35a	4000	10	10	1000	800	8.7979	1.1983	0.42645	1	1
36b	2000	10	10	1000	1000	14.1789	1.4142	0.52	1	1
36a	13000	100	100	13000	13000	12.5226	0.88811	0.25249	1	1
38b	2000	10	10	1000	1000	9.514	0.73735	0.14412	1	1
38a	2000	10	10	1000	1000	14.6502	0.56408	0.55781	1	1
40b	2000	10	10	1000	1000	4.4367	0.38238	0.16234	1	1
40a	1000	10	10	500	500	5.4801	0.49237	0.13876	1	1

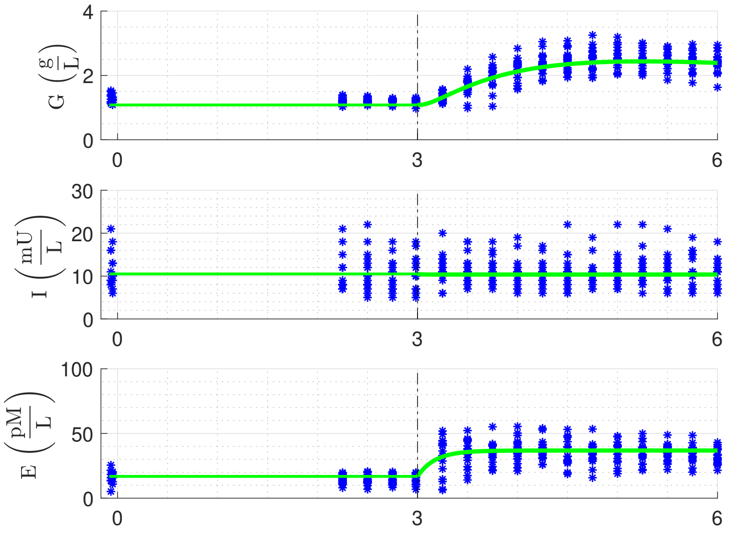

Fig. 6: This plot shows the data points from glucagon challenge test performed on 8 placebo healthy subjects that were not used for fitting. The green lines are the model solution using the average before treatment data reported in Table 7. This plot shows the predictive value of the model.

Table 7: Estimated parameters obtained via smooth profiling method are shown. Every subject has undergone two challenge tests, six weeks apart, one before treatment with drug and one after treatment. The average and the standard deviation of the estimated parameters are also calculated. The last row shows the p-values obtained from comparing the before and after parameters using the Wilcoxon signed rank test. The parameters that are fixed for all subjects are $k_{\text{on}} = 6 \times 10^{-5}$ pmol^{-1}min^{-1}, $k_{\text{off}} = 0.24$ min^{-1} $k_{\text{rec}} = 0.003$ min^{-1}, and $k'_{\text{in}} = K_{ii} = 0$.

	V_1	V_{ii}	V_h	K_{id}	k_{degI}	k_{degE}	k_{in}	K'_1	b_G	V_{id}	$G_{\max}$
Subjects↓	[1/hour]	[1/hour]	[L]	[g/L]	[1/hour]	[1/hour]	[1/hour]	-	[g/(L hour)]	[1/hour]	g/L
30 before	7.2	1.14	4	20	36.6	4.42	18	0.0058	7.69e-09	3.35	2.96
30 After	7.21	0.583	2.81	7.2	38.3	4.53	8.62	0.0111	1.03	2.4	2.15
31 before	3.2	0.788	4.99	15	38.2	4.96	12	0.004	8.4e-14	1.36	2.93
31 After	4.48	1.05	3.07	11.1	37.7	4.78	18.2	0.00507	0.414	1.6	2.02
32 before	4.11	0.831	4.5	11.4	43.4	5.07	12.2	0.00584	4.8e-15	1.42	3.16
32 After	5.6	0.604	3.53	10.5	43.9	4.22	9.85	0.01	0	2.26	2
33 before	4.1	0.695	6	5	30.5	4.05	9	0.01	8.45e-09	0.758	2.18
33 After	7.85	0.907	5	5	28.2	5.01	12	0.015	1.35e-11	0.769	1.42
35 before	6.99	1.44	3.93	4.13	26.9	5.6	17.1	0.00563	2.31e-12	0.663	2.86
35 After	6.33	1.53	5.83	2.86	26.6	5.68	13.6	0.00773	7.87e-12	0.469	2.43
36 before	8.8	0.351	2.89	16.2	20.1	9.78	27.1	0.00319	8.43e-12	2.06	2.63
36 After	10	0.988	5.1	18	22.3	7.72	12	0.012	4.02e-12	2.48	1.75
38 before	4.31	0.954	9.27	14.8	26.7	6.9	20.1	0.00446	0.644	2.11	2.21
38 After	4.9	0.891	6.3	18	25.4	8.34	13.8	0.0072	1.84e-14	2.05	1.83
40 before	6.38	0.973	1.63	1.82	21.5	5.06	56.4	0.00113	3.05e-10	0.446	2.73
40 After	8.1	0.933	8	9.98	24.8	5.79	8.96	0.019	1.39e-11	1.23	2.39
**Before AV**	5.63	0.896	4.65	11	30.5	5.73	21.5	0.00501	0.0804	1.52	2.71
**Before STD**	1.97	0.319	2.28	6.62	8.27	1.84	15.2	0.00257	0.228	0.963	0.353
**After AV**	6.81	0.936	4.96	10.3	30.9	5.76	12.1	0.0109	0.18	1.66	2
**After STD**	1.85	0.294	1.77	5.5	7.93	1.51	3.15	0.0045	0.371	0.768	0.336
**p-values**	0.0391	0.742	1	0.945	0.641	0.844	0.109	0.00781	0.945	0.547	0.00781
**FDR**	0.143	1	1	1	1	1	0.301	0.043	1	1	0.043

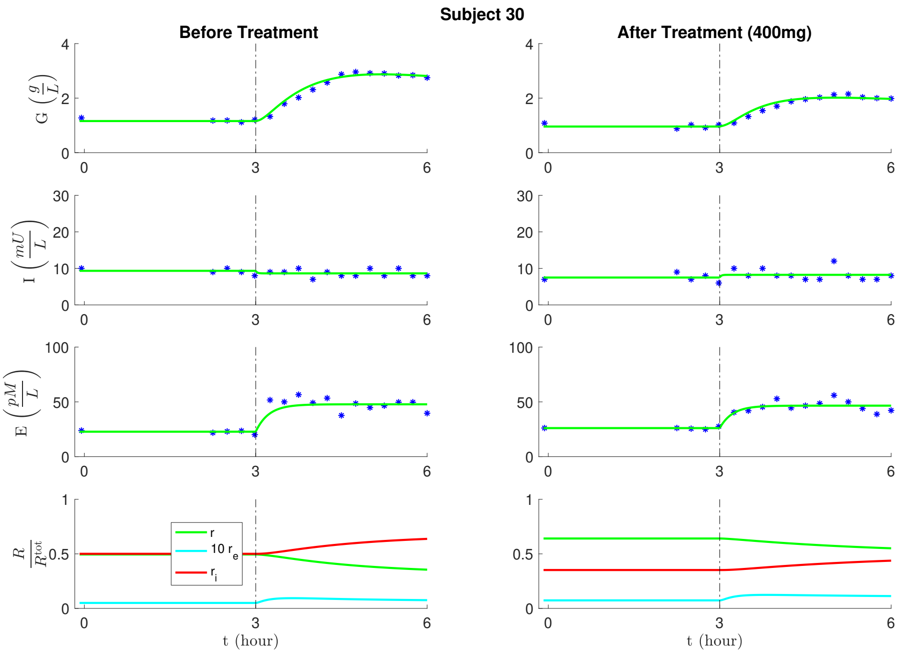

Fig. 7: The result of the fitting for subject 30, before and after treatment with 400mg of the drug.

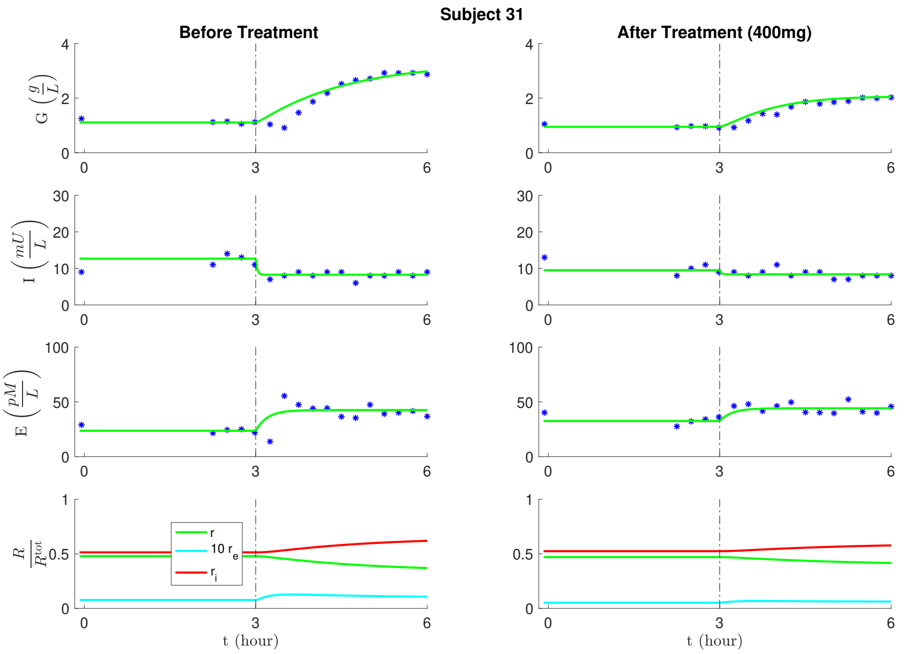

Fig. 8: The result of the fitting for subject 31, before and after treatment with 400mg of the drug.

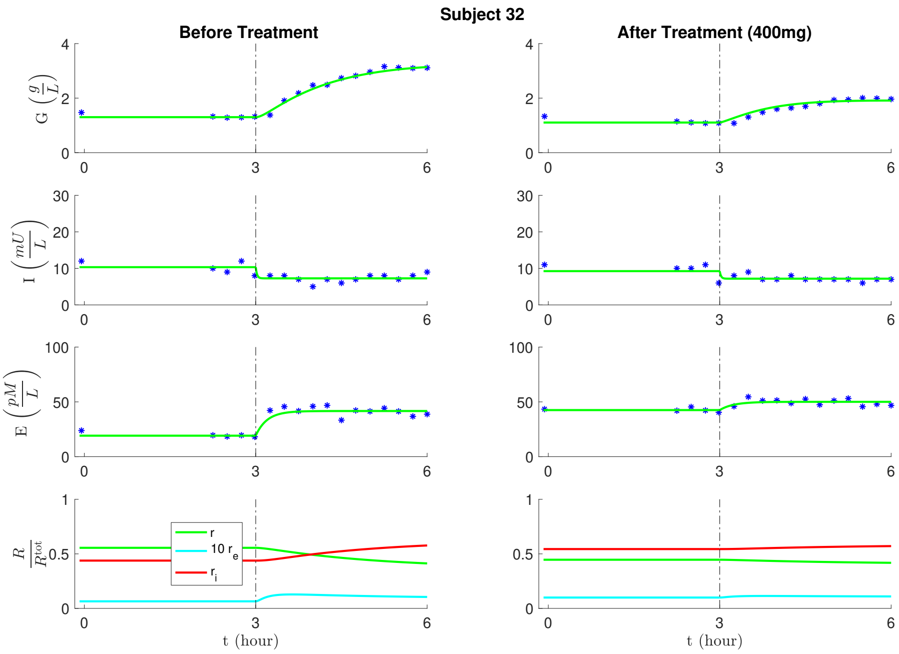

Fig. 9: The result of the fitting for subject 32, before and after treatment with 400mg of the drug.

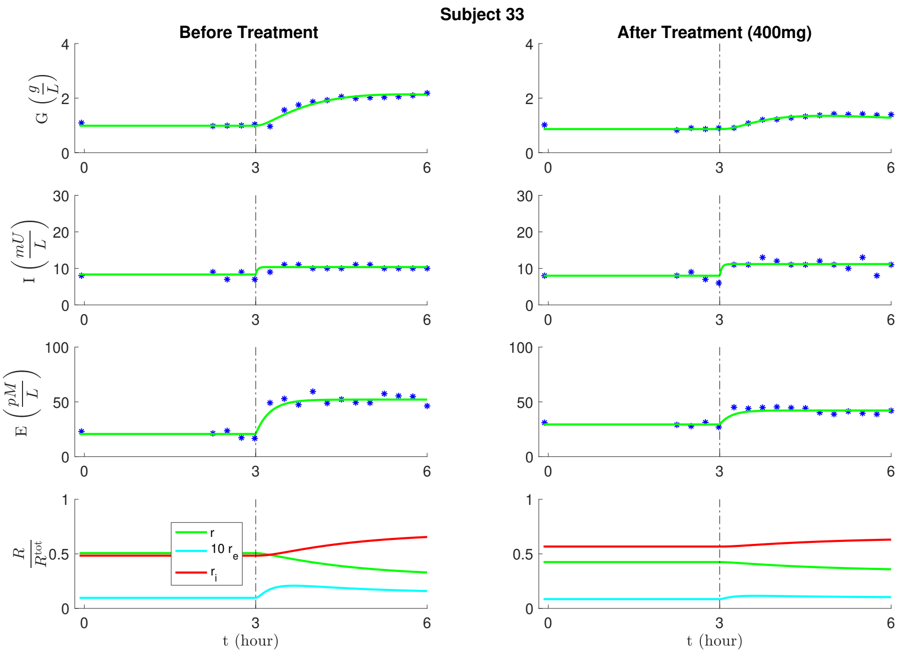

Fig. 10: The result of the fitting for subject 33, before and after treatment with 400mg of the drug.

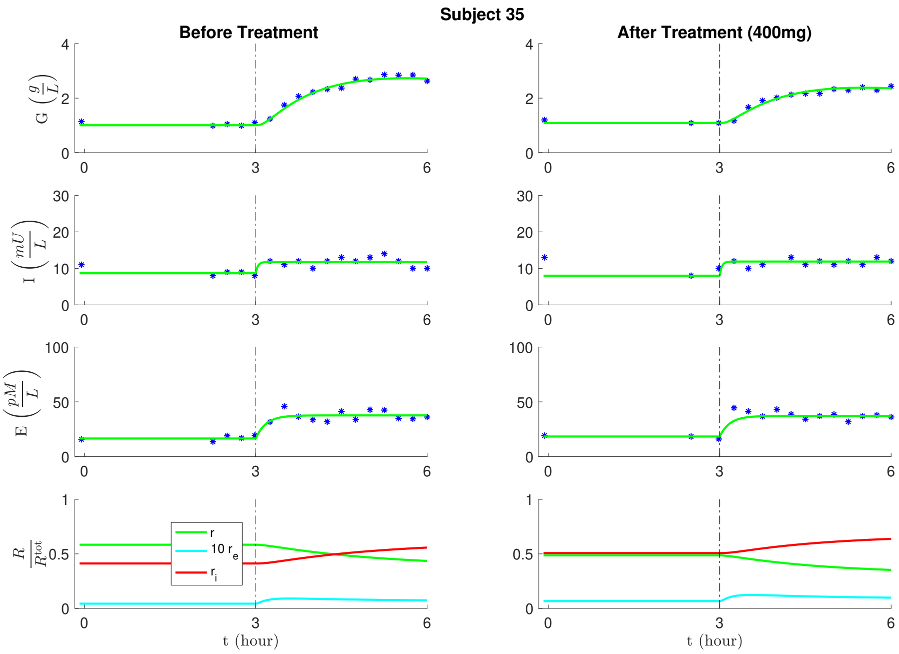

Fig. 11: The result of the fitting for subject 35, before and after treatment with 400mg of the drug.

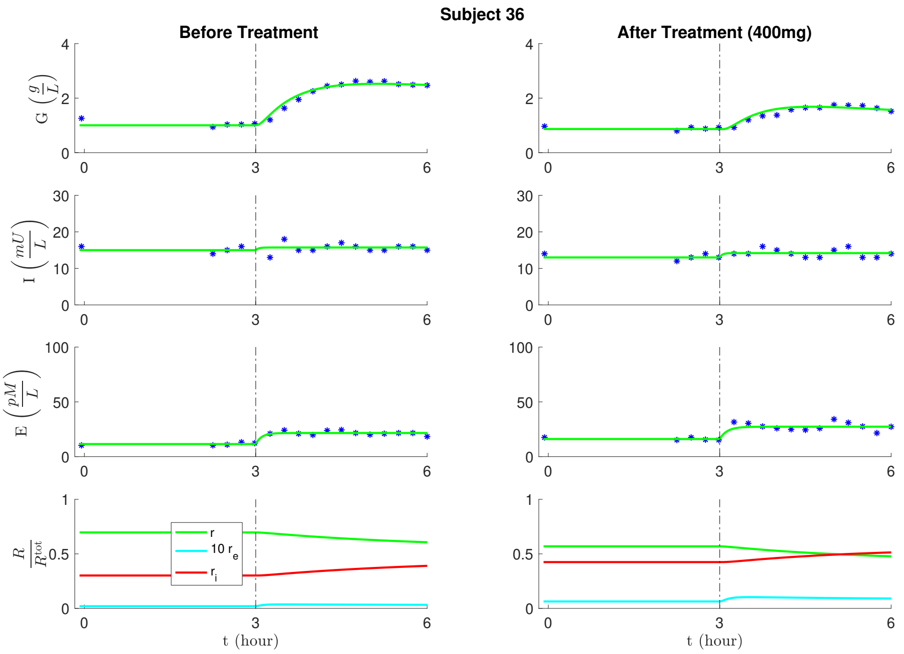

Fig. 12: The result of the fitting for subject 36, before and after treatment with 400mg of the drug.

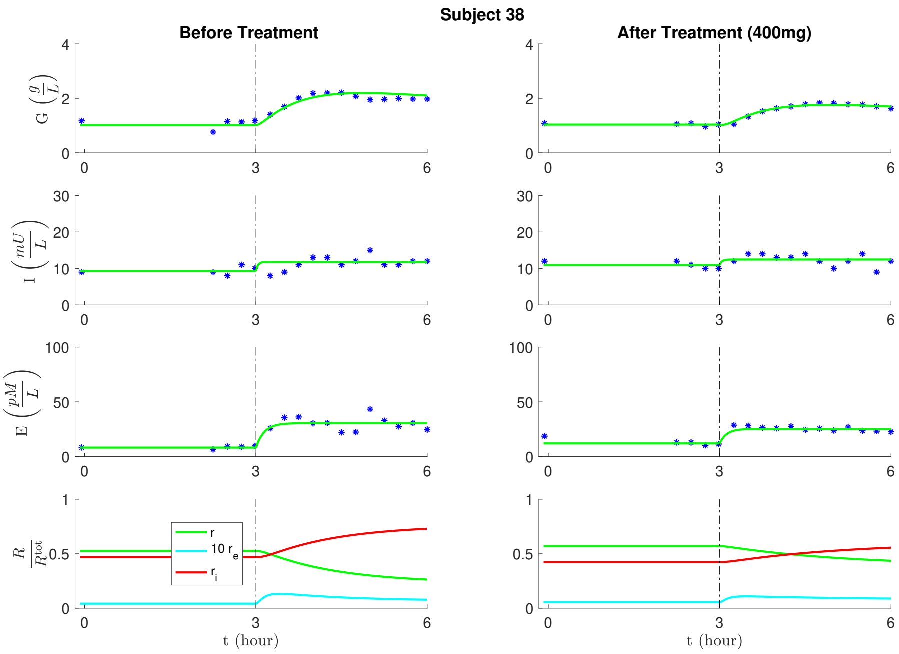

Fig. 13: The result of the fitting for subject 38, before and after treatment with 400mg of the drug.

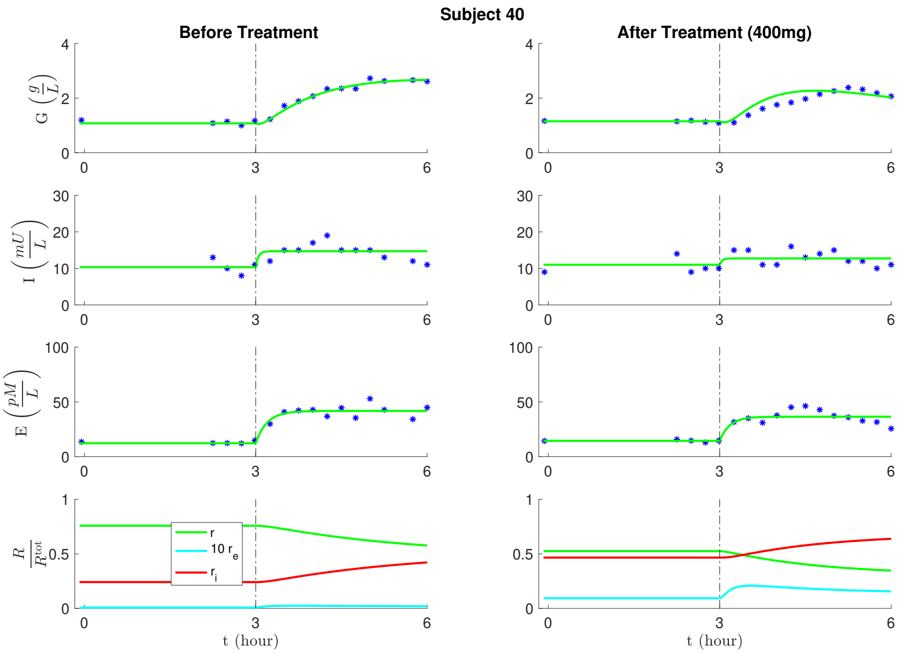

Fig. 14: The result of the fitting for subject 40, before and after treatment with 400mg of the drug.

References

1. Hooker G (2006) Matlab functions for the profiled estimation of differential equations. Cornell University, Ithaca, NY
2. Karlsson J, Anguelova M, Jirstrand M (2012) An efficient method for structural identifiability analysis of large dynamic systems. IFAC Proceedings Volumes 45(16):941–946
3. Ramsay JO, Hooker G, Campbell D, Cao J (2007) Parameter Estimation for Differential Equations: A Generalized Smoothing Approach. J R Statist Soc B 69:741–796, DOI 10.1111/j.1467-9868.2007.00610.x
